# Case report: Development of vanishing bile duct syndrome in Stevens-Johnson syndrome complicated by hemophagocytic lymphohistiocytosis

**DOI:** 10.3389/fmed.2022.975754

**Published:** 2022-10-24

**Authors:** Wan-Chen Lin, Tyng-Shiuan Hsieh, Chia-Yu Chu

**Affiliations:** ^1^Department of Dermatology, National Taiwan University Hospital, Taipei, Taiwan; ^2^Department of Dermatology, National Taiwan University College of Medicine, Taipei, Taiwan

**Keywords:** allergy, non-steroidal anti-inflammatory drugs, drug-induced liver injury, vanishing bile duct syndrome, Stevens-Johnson syndrome, hemophagocytic lymphohistiocytosis

## Abstract

**Background:**

Vanishing bile duct syndrome is a rare drug-induced disease characterized by cholestasis and ensuing ductopenia. Dermatological manifestations of drug hypersensitivity such as Stevens-Johnson syndrome and toxic epidermal necrolysis may also present in such cases. Hemophagocytic lymphohistiocytosis is a hyperimmune response caused by unchecked stimulation of macrophages, natural killer cells, and cytotoxic T lymphocytes.

**Case presentation:**

We report a severe case who presented with concurrent Stevens-Johnson syndrome and vanishing bile duct syndrome complicated by hemophagocytic lymphohistiocytosis after the ingestion of non-steroidal anti-inflammatory drugs. Despite the fact that improvements in vanishing bile duct syndrome can be assumed when combining the clinical lab data clues, as well as repeated liver biopsies showing recovering ductopenia, the patient developed hypovolemic shock combined with septic shock episodes and died on day 236.

**Conclusion:**

To our knowledge, this is the fifteenth report of vanishing bile duct syndrome associated with Stevens-Johnson disease or toxic epidermal necrolysis. Mortality rate remains high without treatment guidelines established due to the rarity and heterogenicity of the population. Further studies are needed to identify possible risk factors, prognostic indicators, and the standard of care for vanishing bile duct syndrome associated with Stevens-Johnson disease or toxic epidermal necrolysis.

## Introduction

Vanishing bile duct syndrome (VBDS) is an uncommon but serious consequence of drug-induced liver injury. It presents clinically as chronic cholestasis and histologically as ductopenia (>50% disappearance of the bile duct in identified portal tracts) (1). Stevens–Johnson syndrome (SJS) is a rare and severe dermatological condition attributed primarily to a T cell-mediated hypersensitivity reaction mostly induced by drugs (2). Hemophagocytic lymphohistiocytosis (HLH) is a life-threatening disorder that may cause multi-organ failure resulting from uncontrolled activation of macrophages, natural killer (NK) cells, and cytotoxic T lymphocytes (3).

We report a rare and difficult case of a 42-year-old male who presented with concurrent SJS and VBDS complicated by HLH.

## Case description

A 42-year-old male with a reported history of resolved hepatitis C was admitted to hospital due to fever and sore throat for 9 days, combined with a generalized rash over the entire body for 5 days.

The patient had not taken new medications in the recent 2 months until 8 days prior to this admission, when the patient visited a local clinic for flu-like symptoms of fever, sore throat, and general malaise combined with muscle weakness and was prescribed non-steroidal anti-inflammatory drugs (NSAIDs)—mefenamic acid and diclofenac—for 3 days with acetaminophen and famotidine. However, the fever persisted despite the treatment, with subsequent development of itchy erythematous macules and papules spreading from the face and trunk to the extremities 5 days prior to this admission. Associated symptoms of ophthalmalgia, conjunctival injection, and lip swelling were also noted. After revisiting the local clinic and obtaining an additional prescription for ibuprofen, he visited the emergency department of another hospital 4 days prior to this admission, where numerous confluent erythematous to purpuric macules and patches on the face, trunk, and extremities, with variously sized erosions arising from the erythematous base were seen. The diagnosis of SJS was thus confirmed. The patient was then transferred to our hospital for further treatment.

**Figure 1:**
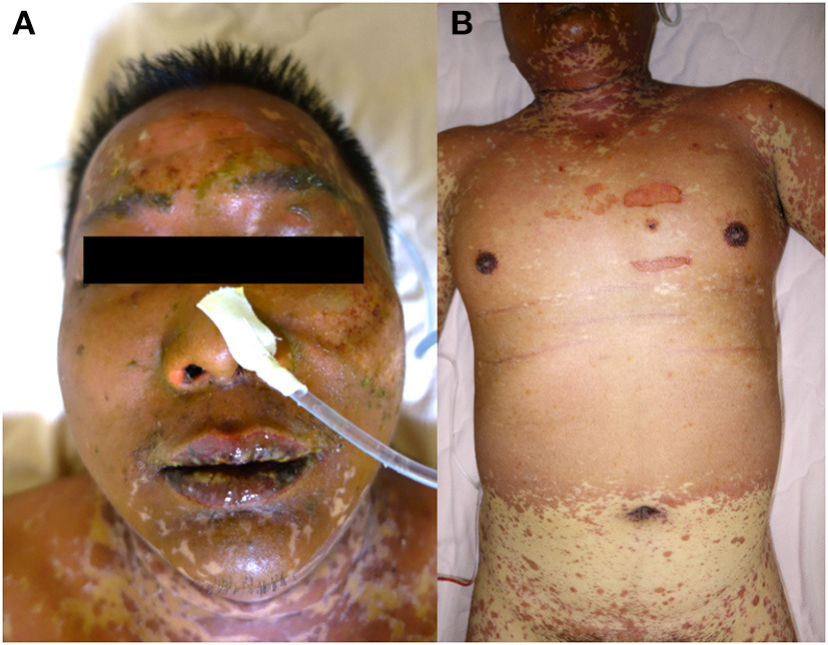
Skin manifestations of a 42-year-old male. **(A)** Diffused erosions covered by crusts on the lips. **(B)** The erythematous vesiculobullous rash had spread to the entire body, along with epidermal detachment.

**Figure 2:**
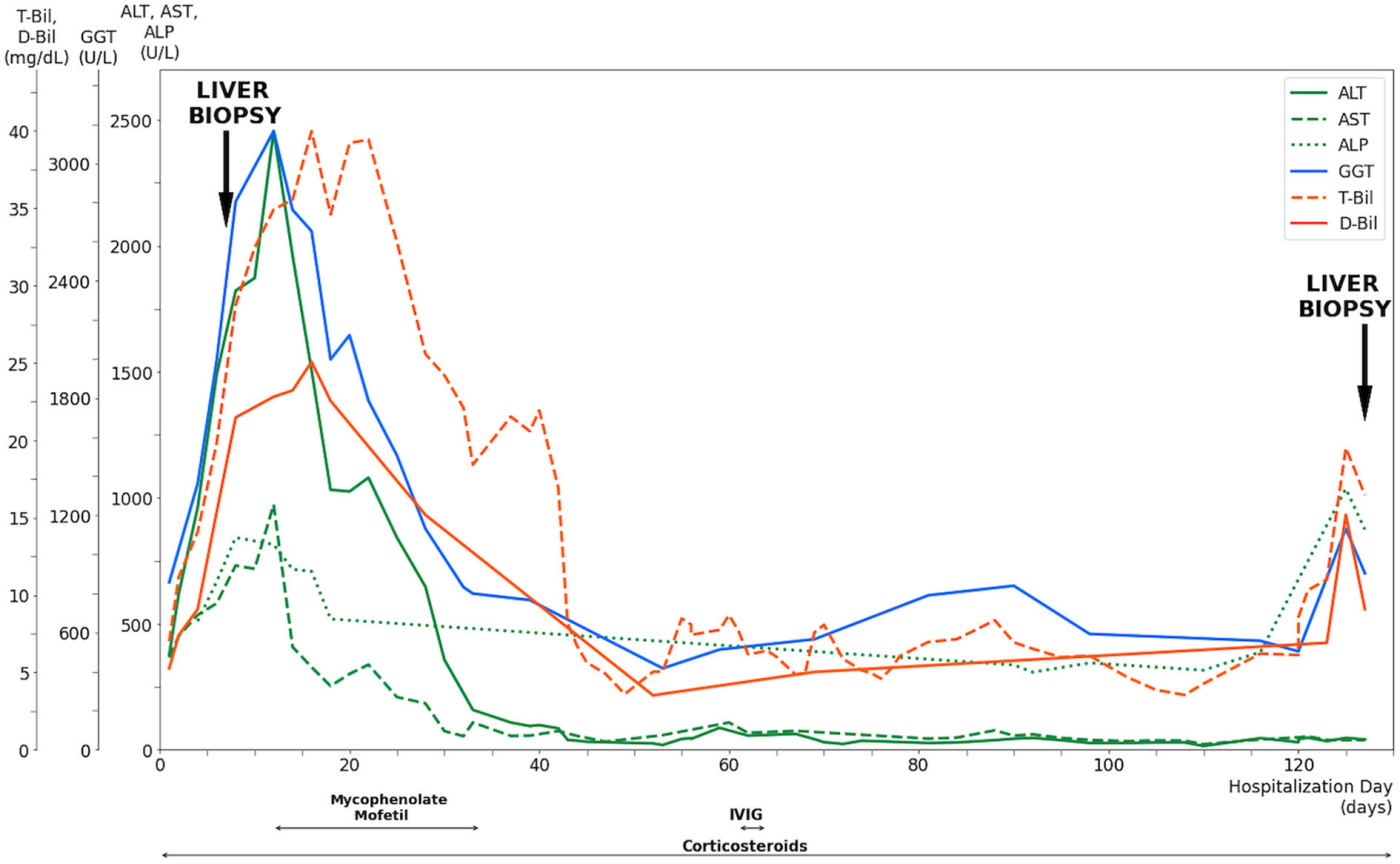
Evolution of serum liver tests and medications of a 42-year-old male after exposure to NSAIDs. Corticosteroids were given since the diagnosis of Stevens-Johnson Syndrome with titration according to the clinical presentation. The progressive elevation of liver enzymes and jaundice had led to a liver biopsy performed on day 6, along with the confirmed diagnosis of vanishing bile duct syndrome. Mycophenolate mofetil was prescribed after the diagnosis while the patient prepared for liver transplantation, which was not performed due to deterioration of clinical conditions. Intravenous immunoglobulin was administered for 3 days after the bone marrow biopsy revealed hemophagocytic lymphohistiocytosis. The liver enzymes and bilirubin levels remained relatively stable until day 125, when significant elevation was observed. Repeated liver biopsy was performed on day 127, which showed vanishing bile duct syndrome in resolution. T-Bil, total bilirubin level; D-Bil, direct bilirubin level; GGT, gamma glutamyl transferase; ALT, alanine aminotransferase; AST, aspartate aminotransferase; ALP, alkaline phosphatase; IVIG, intravenous immunoglobulin.

**Figure 3:**
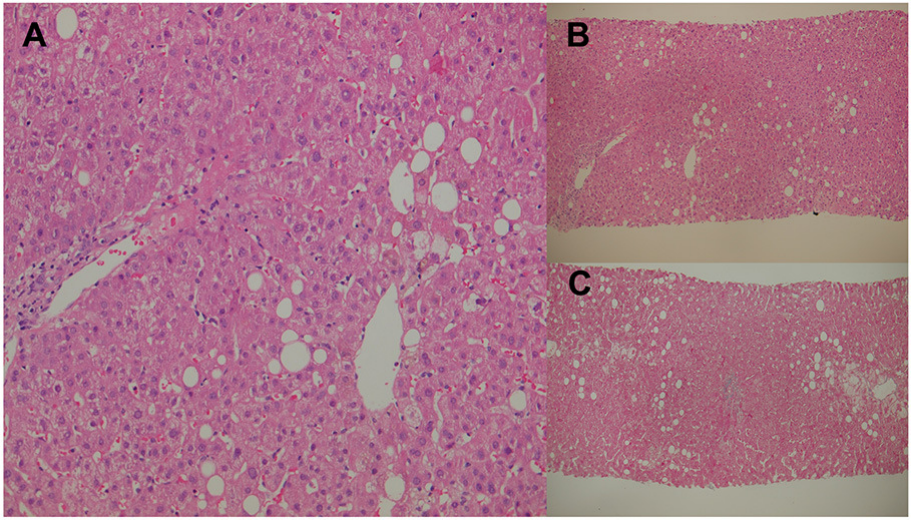
Percutaneous liver biopsies. **(A)** Cholestatic hepatitis with marked perivenular cholestasis and ballooning of hepatocytes seen under high-power field hematoxylin and eosin (H&E) staining. **(B)** Initial liver biopsy showing 10 portal tracts, with only three surviving bile ducts that show degeneration under H&E staining. **(C)** Second liver biopsy showing an increase in the bile duct ratio under H&E staining.

On admission to our hospital, the patient was clear and oriented, with stable vital signs. Physical examinations showed that generalized confluent erythematous to purpuric macules and patches were distributed on the face, trunk, and limbs, with some flat erythematous spots present on the distal extremities. A sum of erythema of 35% total body surface area (TBSA) with blistering of 8% TBSA was calculated after the RegiSCAR review. Mucosal involvement with the presence of conjunctival and oral ulcers were also noted (Figure 1). Viral panel, mycoplasma pneumoniae infection and autoimmune survey yielded negative results (Supplementary Tables S1, S2).The skin lesions gradually improved to brownish and scaly characteristics, with near resolution noted at around day 14 under treatment with systemic corticosteroids (intravenous methylprednisolone), equivalent to 1.2–2.6 mg/kg of prednisone per day, combined with topical antibiotic ointments. Further laboratory examination revealed progressively elevating liver function enzymes and jaundice on day 6 of hospitalization (AST/ALT: 581/1488 U/L; ALP/GGT: 667/1990 U/L; T-Bil/D-Bil: 19.81/15.35 mg/dl) (Figure 2). Elevated IgG, C3, and C4 levels were also noted, without evidence of viral hepatitis. A liver biopsy was thus performed on day 6, and showed cholestatic hepatitis with marked perivenular cholestasis, ballooning of hepatocytes, and Councilman bodies with microgranulomas. Aggregation of histiocytes, neutrophils, and eosinophils were seen in the hepatic lobules. In a total of 10 portal tracts, only three bile ducts survived, which showed degeneration and cell senescence changes (i.e., increased nuclear to cytoplasm ratio, uneven nuclear spacing, drop-out, and syncytia formation; Figures 3A,B). VBDS was thus diagnosed. Due to progression of cholestasis and hepatitis on day 12 (AST/ALT: 973/2455 U/L; ALP/GGT: 815/3165 U/L; T-Bil/D-Bil: 34.91/22.81 mg/dl), systemic corticosteroids were up-titrated to the equivalent of 2.6 mg/kg of prednisone per day, with the capsule form of mycophenolate mofetil initiated at 250 mg twice a day and later up-titrated to 750 mg twice a day. Evaluation for possible liver transplantation was arranged after discussion with the patient and family.

The patient completed pre-transplantation evaluation uneventfully. A significant decrease in liver enzymes and bilirubin levels was observed on day 28 (AST/ALT: 184/647 U/L; GGT: 1,131 U/L; T-Bil/D-Bil: 25.58/15.18 mg/dl). The patient showed resolution of SJS with scars remaining on his extremities and denied discomfort. However, the patient showed a gradual decrease in the hemoglobin level to 8.7 g/dl on day 34, with subsequent discovery of cytomegalovirus (CMV) infection *via* colonoscopy biopsy and blood tests (a CMV viral load of 1,040,000 cp/ml was detected on day 42). Ganciclovir was thus initiated for treatment. Due to the presence of nucleated red blood cells in peripheral blood and the pathological finding of sinus histiocytosis with hemophagocytosis in specimens of the small intestine, a bone marrow study was conducted on day 58. The result showed hemophagocytosis in bone marrow, which met the HLH diagnostic criteria. Intravenous immunoglobulin was thus given from day 61 to day 64.

A repeat liver biopsy was performed on day 127, which revealed intrahepatic cholestasis with marked perivenular bile stasis. Eight portal tracts were observed with seven bile ducts, with six showing degeneration and cell senescence changes. An increase in the bile duct ratio was seen compared to that of the previous study (Figure 3C). Despite the resolution of SJS and VBDS, the patient still developed hypovolemic shock combined with septic shock episodes and died on day 236.

## Discussion

Vanishing bile duct syndrome should be suspected in the clinicopathological setting of persistent cholestasis leading to progressive damage and subsequent loss of intrahepatic bile ducts despite the removal of the offending agent after ruling out other ductopenic diseases (1). Proposed mechanisms of action include direct injury to cholangiocytes after bile excretion, T cell-mediated hypersensitivity reaction resulting in attack against cholangiocytes, and increased exposure to toxic bile salts due to impaired protective defenses of the epithelium. Treatment options remain limited to discontinuation of the offending drug, with possible benefits of ursodeoxycholic acid or corticosteroids when immunoallergic causes are suspected (1).

In our case, which NSAID medication was the exact culprit could not be fully determined due to the prescription of multiple drugs close together. The calculated ALDEN score showed diclofenac as the most likely offending agent, with a score of 3 (4). In reviewing the patient's clinical course, improvement of VBDS could be assumed when combining clinical clues of initially significant decrease in liver enzymes and bilirubin levels after steroid treatment and the repeat liver biopsy on day 127, which showed ductopenia in recovery. However, the patient's general condition further deteriorated, which may partly be attributed to the immunosuppressive effects of steroids and mycophenolate mofetil resulting in CMV and opportunistic infections (5). In addition, steroid-induced poor wound healing may partially account for massive blood passage from resection wounds in the intestine (6).

Hemophagocytic lymphohistiocytosis (HLH) is an aggressive disorder induced by uninhibited activation of macrophages, NK cells, and cytotoxic T lymphocytes, which leads to immune-mediated multi-organ injuries. Clinical and laboratory presentations may vary and include fever, splenomegaly, cytopenia in two or more lineages, hypertriglyceridemia, hyperferritinemia, hemophagocytosis in biopsies, and diminished NK cell activity (3). Epidermal necrolysis (EN) is a lethal mucocutaneous reaction that is mostly medication-induced. EN is further classified as SJS, SJS-TEN overlap, and TEN according to the percentage of detached areas on the skin (7). Due to the similarities between the prodromal phase of EN and infection, the potential viral and bacterial infections should be examined alongside the patient's precise clinical and medication history before the EN diagnosis is finalized. In our case, the patient presented with SJS and VBDS with subsequent development of HLH. T cell-mediated hypersensitivity reaction plays a shared role in the three diseases (1–3), indicating the possible fatality resulting from the hyperimmune response.

To the best of our knowledge, this is the fifteenth report of VBDS associated with SJS or TEN (Table 1). The prognoses varied greatly, with two patients succumbing to death during hospital treatment (15), seven patients requiring liver transplantation (8, 11, 13, 16, 18, 20, 21), and the remaining six patients showing clinical resolution (9, 10, 12, 14, 17, 19). Further studies are necessary to identify the possible risk factors, prognostic indicators, and the standard of care for VBDS with SJS or TEN.

**Table 1:** Drug-induced Vanishing Bile Duct Syndrome (VBDS) associated with Stevens-Johnson Syndrome (SJS) or Toxic Epidermal Necrolysis (TEN).

**Case** **No**.	**Authors**	**Age/Sex**	**Underlying** **disease**	**Culprit drug**	**Interval between** **drug intake and** **VBDS**	**Skin** **lesion**	**Treatment**	**Outcome**
1.	Srivastava et al. (8)	9 y/ F	no known history	ibuprofen	10 days	SJS	UDCA, corticosteroids, tacrolimus	Persistence of jaundice >4 months after onset, patient referred for liver transplantation
2.	Garcia et al. (9)	4 y/ M	mental retardation, cerebral palsy, seizures	carbamazepine	4 months	SJS	UDCA, corticosteroids, tacrolimus	Complete clinical and biochemical recovery within 6 weeks
3.	Taghian et al. (10)	10 y/ F	nickel contact dermatitis, tonsillectomy	ibuprofen	12 days	SJS	UDCA, rifampicin, antihistamine, corticosteroids	Complete clinical and biochemical recovery within 7 months
4.	Karnsakul et al. (11)	7 y/ F	no known history	trimethoprim- sulfamethazole	3 weeks	TEN	corticosteroids, cyclosporin	Persistence of jaundice >10 months after onset, patient awaits liver transplantation
5.	Okan et al. (12)	26 y/ F	no known history	ciprofloxacin	2 weeks	SJS	UDCA, corticosteroids, tacrolimus	Complete clinical and biochemical recovery within 10 months
6.	Juricic et al. (13)	62 y/ F	no known history	azithromycin	1 month	SJS	UDCA, corticosteroids, antihistamine	Persistence of jaundice >7 months after onset, patient received liver transplantation
7.	Kim et al. (14)	7 mo/ F	no known history	ibuprofen	8 days	TEN	UDCA	Complete clinical and biochemical recovery within 4 months
8.	White et al. (15)	6 y/ M	asthma	cefdinir acetaminophen	1 week	SJS	UDCA, corticosteroids, rifampin, plasmapheresis, infliximab	Deceased secondary to respiratory failure
9.	Harimoto et al. (16)	40 y/ F	no known history	acetaminophen	NA	SJS	NA	Persistence of jaundice without specified time range, patient received liver transplantation
10.	Basturk et al. (17)	7 y/ M	no known history	ibuprofen	NA	TEN	UDCA, corticosteroids	Complete clinical and biochemical recovery within 8 months
11.	Momen et al. (18)	52 y/ F	GERD	cephalexin	NA	TEN	NA	Persistence of jaundice >5 months after onset, patient received liver transplantation
12.	Li et al. (19)	6 y/ M	no known history	amoxicillin naproxen	NA	SJS	UDCA, corticosteroids, plasma exchange, traditional Chinese medicine (Pien Tze Huang)	Complete clinical and biochemical recovery within 5 months
13.	Bak et al. (20)	29 y/ F	no known history	pelubiprofen	NA	SJS	corticosteroids	Persistence of jaundice >14 months after onset, patient awaits liver transplantation
14.	Massari et al. (21)	51 y/ F	no known history	ketoprofen	14 days	TEN	corticosteroids, IVIG	Persistence of jaundice >7 months after onset, patient received liver transplantation
15.	Present case	42 y/ M	HCV (resolved)	diclofenac	14 days	SJS	corticosteroids, mycophenolate, mofetil IVIG	Deceased secondary to hypovolemic shock and septic shock.

GERD, gastro-esophageal reflux disorder; HCV, hepatitis C virus; UDCA, ursodeoxycholic acid; IVIG, intravenous immunoglobulin; NA, not available.

## Data availability statement

The original contributions presented in the study are included in the article/Supplementary material, further inquiries can be directed to the corresponding author.

## Ethics statement

Ethical review and approval was not required for the study on human participants in accordance with the local legislation and institutional requirements. The patients/participants provided their written informed consent to participate in this study. Written informed consent was obtained from the individual(s) for the publication of any potentially identifiable images or data included in this article.

## Author contributions

W-CL analyzed the clinical relevant data and drafted the manuscript. T-SH aided in the data collection and edited the manuscript. C-YC provided the resources, supervised the study, edited, and reviewed the final manuscript. All authors contributed to the article and approved the submitted version.

## Conflict of interest

The authors declare that the research was conducted in the absence of any commercial or financial relationships that could be construed as a potential conflict of interest.

## Publisher's note

All claims expressed in this article are solely those of the authors and do not necessarily represent those of their affiliated organizations, or those of the publisher, the editors and the reviewers. Any product that may be evaluated in this article, or claim that may be made by its manufacturer, is not guaranteed or endorsed by the publisher.
