# Supplementary material for: Case report: Development of vanishing bile duct syndrome in Stevens-Johnson syndrome complicated by hemophagocytic lymphohistiocytosis
Source: Front Med (Lausanne). 2022 Oct 24;9:975754. doi: 10.3389/fmed.2022.975754 (PMC9637682; doi:10.3389/fmed.2022.975754)
Supplement: Supplementary file 1 [file Data_Sheet_1.pdf]

Supplementary table 1. Serum viral panel and atypical infection survey results. IgM: Immunoglobulin M; IgG: Immunoglobulin G; EBV: Epstein-Barr virus; EA: early antigen; EBNA: Epstein–Barr virus nuclear antigen; VCA: viral-capsid antigen; CMV: cytomegalovirus; HSV: herpes simplex virus.

Hospitalization Day	Mycoplasma Pneumoniae IgM	Mycoplasma Pneumoniae IgG	EBV EA + EBNA1 IgA	EBV EA IgG	EBV VCA IgG Ab	EBV VCA IgM Ab	CMV IgM	CMV IgG	HSV IgM	HSV-1 IgG	HSV-2 IgG
**Day 2**	Negative	Negative		Negative	Positive	Negative	Negative	Positive	Negative	Positive	Negative
**Day 8**	Negative	Negative					Negative	Positive			
**Day 12**	Negative	Negative		Negative	Positive	Negative	Negative	Positive	Negative	Positive	Negative
**Day 14**			Negative								
**Day 61**				Negative	Positive	Negative					

Supplementary figure I. Timeline of Medication Duration and Dosage. MMF: mycophenolate mofetil; IVIG: intravenous immunoglobulin.

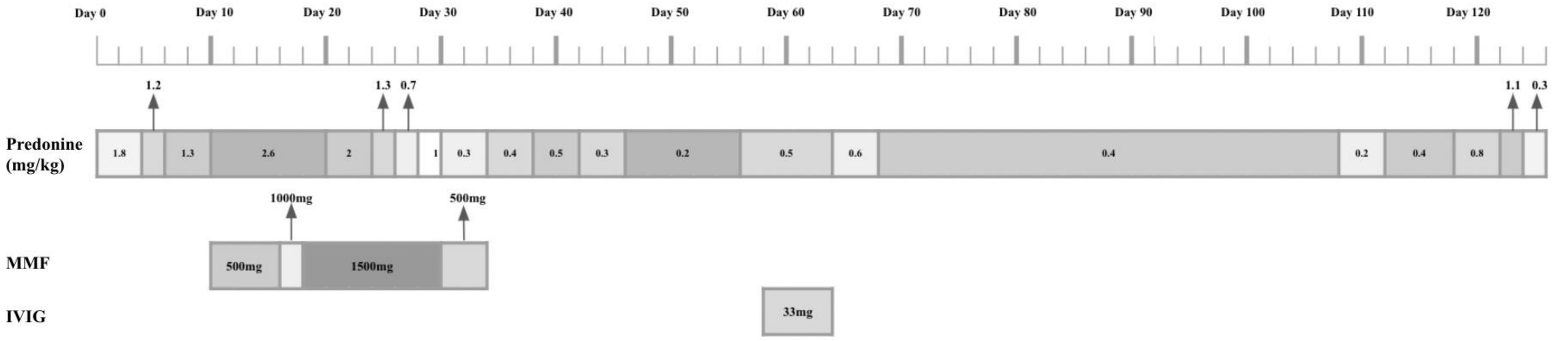

Supplementary table 2. Serum autoimmune survey results. ANA: antinuclear antibody; p-ANCA: peri-nuclear anti-neutrophil cytoplasmic antibody; c-ANCA: cytoplasmic anti-neutrophil cytoplasmic antibody; Anti-ENA: Anti-Extractable nuclear antigen; LKM: liver kidney microsome; EIA: enzyme immunoassay.

Hospitalization Day	ANA	p-ANCA	c-ANCA	anti-ENA (RNP)	anti-ENA (SSA)	anti-ENA (SSB)	anti-ENA (Sm)	anti-ENA (Scl-70)	anti-ENA (Jo-1)	LKM	Mitochondrial antibody	Smooth muscle antibody	Intercellular substance antibody	anti-DNA (EIA) (IU/ml)
**Day 2**	1:320 (+) Homogenous Nuclear													
**Day 5**										1:10 (-)	1:10 (-)	1:10 (-)		
**Day 8**		Negative	Negative	Negative	Negative	Negative	Negative	Negative	Negative					403.89
**Day 63**													1:20 (+)	
**Day 105**	1:40 (-)													<87.48
